# The expert network and electronic portal for children with respiratory and allergic symptoms: rationale and design

**DOI:** 10.1186/1471-2431-13-9

**Published:** 2013-01-16

**Authors:** Kim Zomer-Kooijker, Francine C van Erp, Walter A F Balemans, Bart E van Ewijk, Cornelis K van der Ent

**Affiliations:** 1 Department of Pediatric Pulmonology and Allergology, Wilhelmina Children’s Hospital/University Medical Center Utrecht, PO Box 85090, 3508, Utrecht, AB, The Netherlands; 2 Department of Pediatrics, St. Antonius Hospital, Nieuwegein, The Netherlands; 3 Department of Pediatrics, Tergooi Hospital, Blaricum, The Netherlands

**Keywords:** Asthma phenotypes, Respiratory complaints, Atopic diseases, Follow up study, Children

## Abstract

Data on baseline characteristics of children with asthma to predict individual treatment responses are lacking. We aimed to set up a data-collection system which can easily fill this gap in clinical practice.

A web-based application was developed, named 'Portal for children with respiratory and allergic symptoms', hereafter called Electronic Portal (EP). It contains health- and disease-related questionnaires on respiratory- and allergic diseases. All patients, 1–18 years of age, with respiratory- and/or allergic complaints are invited to enter the EP before their first visit. By using the EP large amounts of data, gathered during routine patient care can be used for research purposes. This may help to further investigate the different treatment related asthma phenotypes and will be helpful to monitor risk factors for other atopic diseases and respiratory infections.

## Background

Asthma is the most prevalent chronic illness in childhood [1]. The prevalence of asthma is ranging from 4 to 12 percent of school age children [2]. A recent study in The Netherlands showed that in a population of 1614 school age children 5% had physician-diagnosed asthma, while an additional 8% had asthma symptoms without knowing to have asthma [3]. Despite advances in the management of asthma in children, it continues to be a condition that has significant impact on children and their families. In a Dutch study both children with diagnosed and undiagnosed asthma had impaired quality of life scores compared to healthy peers and had higher rates of absence from school [4]. The AIRE (Asthma Insight and Reality) study showed only partial effectiveness of asthma care in daily life [5]. In addition, Fuhlbrigge et al. showed that goals of therapy in asthma, based on the National Asthma Education and Prevention Program guidelines, have not been achieved for the majority of children, although more than 70% had mild intermittent disease [6]. The impact of asthma on daily activities is substantial; avoiding exertion (47%) and staying inside (37%) are common approaches to avoid asthma symptoms. These data indicate poor control of asthma in school-age children in affluent countries.

To improve patient care in clinical practice there is an urgent need for predictors of asthma treatment responses. Scarce data are available on predictors of treatment response. Several studies addressed the predictive capacity of family history, clinical symptoms, or lung function parameters for the effect of different treatment regimens. For example, a parental history of asthma or increased levels of exhaled Nitric Oxide (eNO) might predict a beneficial effect of ICS [7-10] while in adults LTRAs might be especially beneficial in asthma patients who smoke [11]. In cases where group-wise differences between different therapies are lacking [12,13], predictive baseline characteristics might be helpful to predict which therapy has the best risk-benefit ratio in the individual child.

The evaluation of the predictive capacity of comprehensive clinical and laboratory parameters for treatment responses requires analysis of a large and diverse patient population from different clinical settings and prospective follow-up. Recently, we started an extensive nationwide study in The Netherlands to compare different treatment strategies for children with respiratory and allergic symptoms and to evaluate predictors of treatment responses. In a strongly internet-supported network of academic and general pediatricians in The Netherlands (the ‘Expert Network’) large numbers of patients are recruited and evaluated using an Electronic Portal. Here we aim to describe the design of both the Expert Network and the Electronic Portal.

## Methods

### Study design

The Electronic Portal (EP) is used by the members of the Expert Network (EN) as a clinical tool to prospectively collect data in children with respiratory and allergic symptoms. The EP is used firstly to thoroughly screen patients on the presence of certain symptoms and possible risk factors, before their first outpatient department-visit. Secondly, patients can be followed-up on a regular basis without intervention of their caregivers. At start uniform information about atopic diseases, respiratory infections, exposure to potential toxins, and demographic information is collected by the patients. Afterwards data on treatment, disease control and treatment effects are monitored. In this way pre-treatment patient characteristics can be related to treatment and disease outcomes. Recruitment and follow up of children started in June 2011.

### The expert network

**Figure 1:**
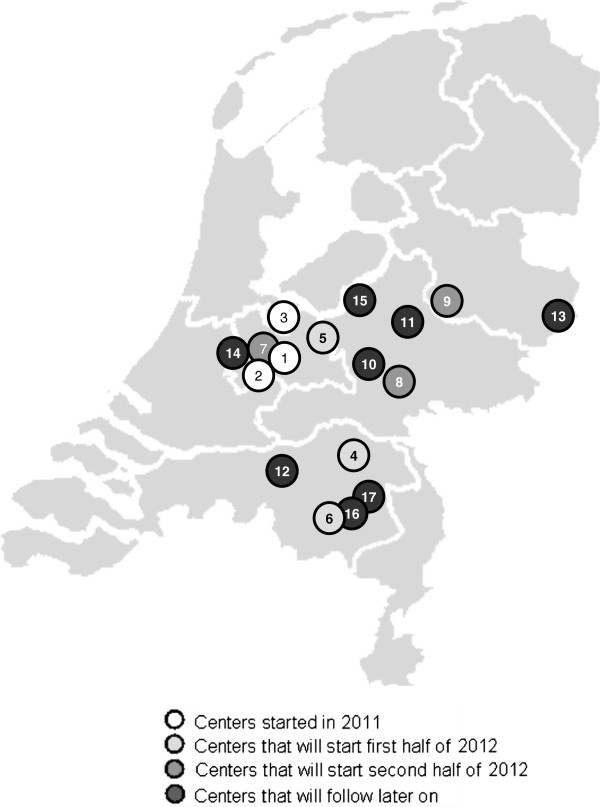
**The Dutch Expert Network.** 1 = Utrecht, 2 = Nieuwegein, 3 = Blaricum, 4 = Oss, 5 = Amersfoort, 6 = Veldhoven, 7 = Utrecht, 8 = Arnhem, 9 = Deventer, 10 = Ede, 11 = Apeldoorn, 12 = Tilburg, 13 = Enschede, 14 = Woerden, 15 = Harderwijk, 16 = Helmond, 17 = Eindhoven.

In a nationwide collaborative network of Dutch caregivers at least 3000 children presenting with asthma symptoms will be included from June 2011. The EN consists of caregivers in the primary-, second- and third line health care. The members of the EN are general practitioners, pediatricians and specialized pediatricians in pulmonology, allergology, dermatology, infectiology and otolaryngology. We aim to include at least 15 large pediatric clinics (for current status see Figure 1).

Members of the EN are personally instructed how to use the EP. The EN has three-monthly meetings in which data from the EP are analyzed and compared between centers. Information about meetings, diagnostic and treatment protocols, and scientific updates on atopic diseases can be found on a supporting website.

Children between the ages of 0–18 years, referred to a member of the EN because of respiratory- or atopic complaints are eligible to participate and are asked to participate in the EP. Also known patients are eligible to participate in the EP. Each centre has its own account. With this, access is given to the data of their own patients, and records can be made and printed with results per patients. Patients with congenital pulmonary defects or cystic fibrosis are excluded. Also (parents of) patients who do not understand the Dutch language will be excluded, however, if children above the age of 11 do understand the Dutch language well, they are eligible to participate themselves. Informed consent for use of the questionnaires and clinical information is given by an electronic check mark. The medical ethics committee of the University Medical Centre Utrecht has approved the protocol.

### The electronic portal

**Figure 2:**
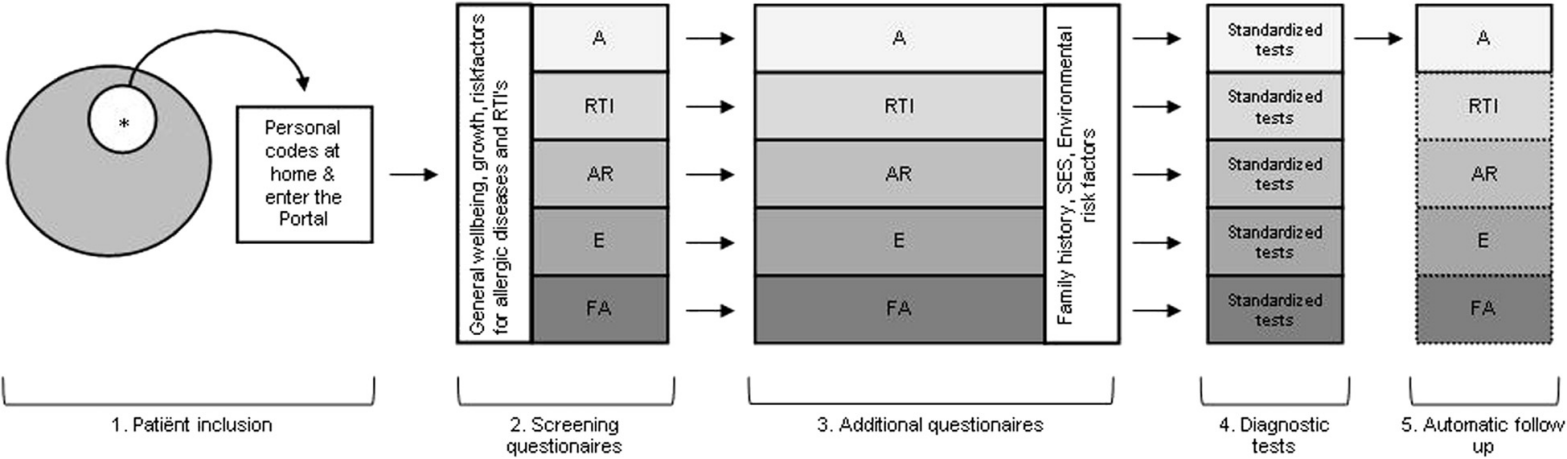
**Structure and way of usage of the Electronic Portal.** * = Patient consulting one of the EN members, A = Asthma, RTI = Respiratory Tract Infections, AR = Allergic Rhinoconjunctivitis, E = Eczema, FA = Food Allergy,··· To be developed.

The Electronic Portal is a web-based application developed by the University Medical Centre Utrecht, in collaboration with Vital Health software. The EP can be approached via the url http://www.luchtwegportaal.com. The supporting website presents information on three levels: for the patient, the parents, and the members of the Expert Network, and contains disease information, information on the EP, and protocols for physicians. From this website the EP can be entered with a unique personal code. The information in the EP consists of personal patient information, validated questionnaires, diagnostic test results, and an automatic follow up function. Individual data in the EP are accessible for both the patient and his caregiver and structured reports can be generated on screen and on paper. The content of the EP is summarized in Table 1. Three age-dependent questionnaire sets are available in the EP, and are automatically selected based on the age of the child; a set for children 0–1 years, one for children between 1–11 years and a set for children above 12, in which most of the questions are directed to the child itself. The structure of the EP, and the following order in which the EP is used is shown in Figure 2.

**Table 1:** Content of the Electronic Portal for children with respiratory and allergic symptoms

**1. Screening Part**	**Includes**
Personal data	DOB, weight at birth, development, vaccination status
General Health Status	RAND questionnaire
General medical history questions	Known risk factors for atopic diseases
Screening questions on atopic and infectious diseases	ISAAC core questions and non-validated questions
**2. Additional Part**	
Asthma	
Symptoms	ISAAC additional questions, ACT, medication use
Treatment compliance	MARS
Quality of life	PAQoL
Infections	
Symptoms	Non-validated questionnaire
Quality of life	OM-6
Allergic Rhinoconjunctivitis	
Symptoms	ISAAC additional questions, ARIA, medication use
Quality of life	RQLQ
Food allergy	
Symptoms	Non-validated questionnaire
Quality of life	FaQoL
Eczema	
Symptoms	SA-EASI
Quality of life	IDQL or CDLQI
**3. Diagnostic test results**	
Lung function tests	FEV1, NO, BDR or Methacholine challenge test
Laboratory results	Inhalation screening (sIgE)
Allergy test results (when applicable)	SPT, Food challenge results
**4. Follow-up Part**	
Treatment	Medication use
Symptom control	ACT
Treatment compliance	MARS scale
Lung function	FEV1, NO, BDR or Methacholine challenge test

*DOB* = Date of Birth.

*FEV1* = Forced Expiratory Volume in 1 second.

*NO* = Nitric Oxide.

*BDR* = Bronchodilator response.

*SPT* = Skin Prick Test.

For abbreviations concerning questionnaires: see Table 2.

### Baseline examination

#### Screening questionnaires

After entering the EP, parents are asked to fill in screening questionnaires which aim to screen on the presence of atopic diseases. Core questions of the ISAAC questionnaire on asthma, allergic rhinoconjunctivitis and eczema are used for this purpose. In addition, questions about respiratory infections and food allergy are included. Based on the answers in the screening part, additional specific questionnaires on each disease topic are selected or not, to be filled in subsequently. Information about growth parameters, breast feeding and vaccination status are obtained from personal health care files by the parents. This health care file is a document that every child in the Netherlands owns and is used in the primary care setting during the first years of live. The general health status is determined based on the RAND questionnaire (Table 2). The screening questionnaires also contain questions about known risk factors for infections (as use of a consoler, day care) and atopic diseases (as smoking, pets, and breastfeeding).

**Table 2:** Questionnaires in the additional part of the Electronic Portal

**Questionnaire**	**Description**	**Score range**
RAND GHRI [14,15]	7-item general health questionnaire. Developed for use in children 0,5-12 years of age	Range: 7-32
		32 = good health
C-ACT [16]	7-item questionnaire. Developed to measure asthma control in children 4–11 years of age. 4 questions are for the child, 3 for the parent.	Range: 0-27
		≥ 20 = well controlled
ACT [17]	5-item questionnaire developed to measure asthma control in children ≥12 years.	Range: 5-25
		≥ 20 = well controlled
MARS [18]	9-item questionnaire, developed to measure medication adherence.	Range 0-5
		Mean score >4.5 = ‘adherent’
PAQLQ [19]	23-item questionnaire, in 3 domains. Developed to measure asthma-specific health-related QoL in children 6–18 years of age.	Range 0-7
		higher scores indicate better QoL
Brouilette score [20]	3-item questionnaire to assess presence of OSAS	> 3,5: OSAS present
		- 1 to 3.5: uncertain OSAS
		< −1: OSAS not present
OM6 [21]	6-item questionnaire in 6 domains. Developed to measure change in ear-related handicap in children with recurrent acute otitis media and otitis media with effusion	Range 0–7 (mean)
		7= severe
ARIA [22]	5-item questionnaire, developed to measure presence and severity of rhino-conjunctivitis	Classification into: intermittent or persistent rhinitis; and severity: mild or moderate/severe
PRQLQ [23]	23-item questionnaire in 5 domains. Developed to measure the functional problems in rhino-conjunctivitis in children 6–12 years of age	Range: 0–6 (mean)
		6 = maximal impairment in health related quality of life
AdolRQLQ [24]	25-item questionnaire in 6 domains. Developed to measure the functional problems in rhino-conjunctivitis in children 12–17 years of age	Range: 0–6 (mean)
		6 = maximal impairment in health related quality of life
FAQLQ-CF [25]	24-item questionnaire, in 4 domains. Developed to measure food allergy related QoL in children 8–12 years of age	Range: 1–7 (mean score)
		7 = maximal impairment in health related quality of life
FAQLQ-TF [26]	23-item questionnaire, in 3 domains. Developed to measure food allergy related QoL in children 13–17 years of age	Range: 1–7 (mean score)
		7 = maximal impairment in health related quality of life
SA-EASI [27,28]	10-item questionnaire. Developed to measure the caregiver's self-assessment of the severity of his/her child's atopic dermatitis	Range: 0–72 (acute score)
		72 = very severe
IDQL [29]	10-item questionnaire. Developed to measure < 4 years of age	Range: 0-30
		higher score means larger impairment of QoL
CDLQI [30]	10-item questionnaire. Developed to measure 4–16 years of age	Range: 0-30
		higher score means larger impairment of QoL

*RAND GHRI*, RAND General health rating index.

*C-ACT*, Child-Asthma Control Test.

*ACT*, Asthma Control Test.

*MARS-9*, 9-item Medicine Adherence Rating Scale.

*PAQLQ*, Paediatric Asthma Quality of Life Questionnaire.

*OM6*, 6-item Otitis Media questionnaire.

*ARIA*, Allergic Rhinitis and its Impact on Asthma.

*OSAS*; Obstructive Sleep Apnoea Syndrome.

#### Additional questionnaires

The aim of the additional questionnaires is to extensively explore the complaints of the patient, his medication use and habits, and measure the disease related quality of life. Details of the supplementary questionnaires in the EP, and the meaning of the corresponding scores are given in Table 2. Questionnaires about asthma, respiratory tract infections, allergic rhinoconjunctivitis, eczema and food allergy are included. In addition to the questionnaires mentioned in Table 2, additional questions about asthma and rhinoconjunctivitis are included [31]. Besides disease specific questionnaires, information on environmental factors, pet exposure, (parental) smoking and social economic status are obtained, partially adopted from the ISAAC questionnaire [32].

### Diagnostic tests

Caregivers from the EN can add results of diagnostic tests to the EP. Protocols are written to ascertain uniform performance of different tests.

#### Respiratory function

In all new patients suspected for asthma, lung function and allergy tests are performed according to the Dutch national guidelines [33]. Spirometric assessments, e.g. maximal flow-volume curves, are measured according to the ATS/ERS standards [34]. The highest values of three correctly performed manoeuvres are used for analysis. Recorded parameters are FEV1 (Forced Expiratory Volume in one second) and FVC (Forced Vital Capacity). To measure the bronchodilator response 800 microgram of salbutamol is administered via a metered dose inhaler using a volumatic spacer (GSK, Uxbridge, UK). Airway reversibility is defined as an increase of FEV1 of ≥ 12% of the predicted value 10 minutes after administration of salbutamol.

Bronchial hyper responsiveness (BHR) is assessed by a challenge with nebulized methacholine according to the ERS/ATS guidelines [35]. All children will be asked to withhold from taking rescue medication for at least 12 hours, and long acting beta two agonists at least 24 hours beforehand. A child will be defined as having BHR when FEV1 has dropped by ≥20% from baseline during the inhalation challenge. In children with a baseline FEV1 ≤70% no challenge will be performed.

In all known patients with asthma spirometry assessment (a bronchodilator response (BDR) or on indication a challenge test) is annually performed, according to the national guideline [33].

#### Other test results

Depending on the situation of the patient, more diagnostic tests may be performed when this is considered necessary for patient care by the physician. For instance, in a child presenting with recurrent infections initially a culture may be taken and lab tests to assess the immunologic status may be performed, before a lung function test will confirm the diagnosis of asthma. The EP does offer the opportunity to enter those test results in the system in a structured way. Cultures (nasopharyngeal, sputum, ear, nose) and lab results in case of suspicion of a immune deficiency can be registered when applicable. Atopic test results, such as an ImmunoCAP for food allergens or inhalation allergens, food challenge results or skin prick test results can be entered. Test results can be filled in on predefined schedules. Also the doctors-diagnosis will be entered in the EP, and other diagnoses can be entered over time.

### Follow up and study endpoints

By activating the follow up function in the EP, patients are notified by email that a short questionnaire is ready to be filled in by parents and/or patient in the EP at predefined 3-month intervals, which is once every season. (content: see Table 1 section follow-up). In order to obtain a validated measure of asthma control, the EP uses the validated C-ACT, or ACT, depending on the age of the child. Adherence to treatment is assessed by using the Medication Adherence Report Scale (MARS) comprising questions on medication use behavior and adherence [36]. Medication use is registered by parents.

## Privacy

The handling of personal data complies with the Dutch Personal Data Protection Act. All data are stored in a large database, which is maintained by Vital Health Software. Storage and protection of the data is performed according to the NEN 7510 guideline. Privacy is protected by encrypted storage of personal information in the database. Exchange of data is protected by a security protocol to prevent damage, loss, unauthorized access or abuse of data. The EP can only be accessed with personal access codes.

The EP offers different user levels. Each level has its own function and privileges, such as a professional (to give access to the EP to patients, and to view their own recruited data), an application manager (to give access to the EP to professionals; access to all processes and modules, including the databases), and patient (access to their own data). Each participating centre has its own access codes, and data from other centers cannot be seen or modified.

## Results

### Recruitment

At the time of writing 1500 children have been invited to participate, of whom the baseline questionnaire has been completed in 740 (49%) patients. 478 patients were selected to be followed up based on a diagnosis of a recent asthma diagnosis or new symptoms that were assigned to asthma by the pediatrician. Recruitment has been underway for 1 year in 3 centers (Figure 2), for 5 months in 2 centers and 2 months in one center. Two other centers have confirmed participation in the study, and will start at the end of 2012 with inclusion.

## Discussion

In current clinical practice large amounts of data are gathered during routine patient care. Very little of these data are available for research purposes because data are not recorded in a structured way. Here we describe an EP which facilitates the EN to collect data in a structured way with minimal effort of the caregivers themselves. This EP offers several opportunities.

Since the start of inclusion, in June 2011, 1500 patients were invited to participate. At present 740 patients (49%) have completed the baseline questionnaire. Most patients that have not completed the questionnaire are known asthmatic patients that visit their doctor once per year. These patients will fill in the questionnaire shortly before their next doctor visit. In 95% of the cases informed consent was given to use EP-data for research purposes. This shows the EN is able to gather a large number of patients within a relative short period. As a result a large database will be available within a relatively short time.

Large population based observational studies, mainly birth cohort studies, have been published and mainly studied determinants of asthma [37-39]. These data are not suitable to study treatment related asthma phenotypes of asthma in children (e.g. treatment response to inhaled steroids in asthmatic patients with eczema, compared to those without eczema; or treatment response to long-acting beta-agonists in asthmatic patients with marked airway reversibility compared to those without (or with minor) reversibility); firstly because of the small number of patients with asthma in most of these studies. Although birth cohorts may be large, asthma may be present in about 5% of the children above the age of five. The number of patients using asthma medication on regular basis, which is only a sample of this 5%, does not allow comparing therapy response within the different treatment regimens. Especially in a heterogeneous disease such as asthma, large patient numbers are needed to explore those treatment defining phenotypes.

Strict inclusion criteria are used in randomized trials to study the efficacy of treatment trials. The outcomes of those studies are applicable to this selected group, but difficult to generalize in the heterogenic asthmatic population seen in daily practice. The EP enables collection of data gathered during daily practice of an unselected population with asthma (and other atopic diseases), for research purposes. By including large samples of patients, the outcomes will be usable in daily practice. Data from the EP will be used to study the effectiveness instead of the efficacy, which makes the outcomes more applicable in daily practice.

Currently, the automatic follow up function is enabled for asthmatic patients only. However, this function will be available at the end of this year for the other disease topics included in the EP: allergic rhinoconjunctivitis, eczema, food allergy and (upper and lower-) respiratory tract infections.

Apart from the research relevance, the patients participating in the EP will be followed up in time, which means that their complaints will be monitored actively by the EP without extra effort from the doctor. In regular asthma care, the frequency of visits is often once per year in stable periods. During this visit it may be difficult for parents and patient to recall how the last 12 months have been. The EP makes it possible to have a whole year through-overview of asthma control, medical treatment response and medication use for the doctor, as well as for the patient. Transparency in hospital care is also increased by access to their test results in the electronic EP by each individual patient, which may increase the involvement of the patient in his treatment [40].

The EP supports a more structured way of working within the collaborative network. This may support the use and implementation of national guidelines on atopic diseases. Each participating hospital creates its own patient database. With this database the performance of each centre can be monitored and compared to other centers. Furthermore, working strategies or other knowledge can be exchanged to improve daily practice within the centers.

Due to the use of a web based application, there will be a selection in the population that is included in the EP. Currently in the Netherlands, 1% of all persons between 11–45 years of age do not have access to internet at home [41]. The main reason for not having internet-access is ‘no interest’. Because financial reasons seem to play a much smaller role, this will probably not lead to a selection in our patient group (in social economic state). However, also a good understanding of, and ability to read the Dutch language is an inclusion criterion. This will lead to a selection of patients, because the 1.5 million functional analphabetic persons in the Netherlands will mainly evolve within the lower social economic class. One third of those persons are foreigners [42]. How large this selection is will be analyzed.

We conclude that the use of current web-based services like the described EP can be helpful to support extensive data collection in Expert Networks.

## Abbreviations

ACT: Asthma Control Test;AIRE: Asthma Insight and Reality;BDR: Bronchodilator Response;BHR: Bronchial Hyperresponsiveness;C-ACT: Child-Asthma Control Test;EN: Expert Network;eNO: Exhaled Nitric Oxide;EP: Electronic Portal;FEV1: Forced expiratory volume in one second;FVC: Forced Vital Capacity;ICS: Inhaled corticosteroids;LTRAs: Leukotriene receptor antagonists;MARS: Medication Adherence Report Scale

## Competing interests

WB is a member of the pediatric medical advise committee of GSK and receives an attendance fee; For organizing the asthma course (GSK) for pediatricians he receives an organizing fee. All other authors have no conflicts of interest to declare. No Financial support was provided.

## Authors’ contributions

KZK is the primary investigator and responsible for data collection and analysis and for drafting the manuscript. CKE has designed and supervised the study, and FCE, WB and BE have contributed to the draft of the manuscript and collection of the data. All authors read and approved the final manuscript.

## Pre-publication history

The pre-publication history for this paper can be accessed here:

http://www.biomedcentral.com/1471-2431/13/9/prepub
